# Adaptation for Regulatory Application: A Content Analysis of FDA Risk Evaluation and Mitigation Strategies Assessment Plans (2014–2018) Using RE-AIM

**DOI:** 10.3389/fpubh.2020.00043

**Published:** 2020-02-25

**Authors:** Gita A. Toyserkani, Linda Huynh, Elaine H. Morrato

**Affiliations:** ^1^Food and Drug Administration, Silver Spring, MD, United States; ^2^Oak Ridge Institute for Science and Education (ORISE) Program, Silver Spring, MD, United States; ^3^Colorado School of Public Health, University of Colorado Anschutz Medical, Aurora, CO, United States

**Keywords:** RE-AIM, REMS, FDA, risk management, regulatory science, drug safety, program evaluation, implementation science

## Abstract

**Background:** Risk Evaluation and Mitigation Strategies (REMS) are safety programs that U.S. Food and Drug Administration can require to ensure a drug's benefits outweigh its risks and can be considered public health interventions. FDA's 2019 *draft Guidance for Industry on REMS Assessments* encourages the development of “novel methods for assessing REMS [to] help advance the science of post-market assessment of effectiveness of risk mitigation strategies.”

**Objective:** To characterize REMS assessment plans using RE-AIM (Reach, Effectiveness, Adoption, Implementation, Maintenance) framework and identify areas for advancing methods for evaluating REMS programs. RE-AIM was selected for its wide application evaluating the translation of scientific advances into practice for public health impact.

**Methods:** A content analysis of REMS assessment plans (*N* = 18) and measures(*n* = 540) was conducted for REMS programs approved by FDA between 1/1/2014–12/31/2018. Eligibility criteria were: a new drug application or biologic license application, included FDA-mandated mitigation strategies called elements to assure safe use (ETASU), and represented a single product REMS program. Assessment plans were collected from publicly available regulatory approval letters from REMS@FDA website. Blinded reviewers categorized each REMS assessment measure to a RE-AIM dimension, adjudicated their application (average IRR 75%), and refined the adapted dimensions' definitions. Dimensions were also mapped to *REMS Assessment guidance* categories.

**Results:** The median number of assessment measures per REMS assessment plan was 31 (IQR: 21–36). Frequency of measures per RE-AIM criteria per REMS program was: Reach (median = 2; IQR: 2–4); Effectiveness (median = 2.5; IQR:1–4); Adoption (median = 3.5; IQR: 2–5); Implementation (median = 18; IQR: 15–24); Maintenance (median = 0; IQR: 0–1). Adoption (among prescriber, health system agents of implementation) was more commonly assessed than Reach (population-attributable number of patients affected). Assessment of heterogeneity of Adoption and Reach was generally absent. Implementation assessment measures were most common among drugs requiring evidence of safe-use conditions before dispensing or administering the drug. Patient-level Effectiveness and Maintenance assessments were most common among drugs requiring patient monitoring.

**Discussion:** Implementation science frameworks, such as RE-AIM, can be applied to characterize REMS assessment measures and identify opportunities for standardizing and strengthening their evaluation. Methods to measure Maintenance are needed to provide real-world evidence of REMS integration into the healthcare system.

## Introduction

The U.S. Food and Drug Administration (FDA) is responsible for protecting the public health of Americans by assuring the safety and efficacy of human drugs and biological products (1). Over the past two decades, modernization of post marketing drug safety and risk management has received increasing attention (2, 3). Post marketing safety issues include serious adverse events, product quality issues, and medication errors (4). Given the U.S. population's large and increasing magnitude of medication exposure, the potential for harms from adverse drug events constitutes a critical patient safety and public health challenge. An estimated one-third of all hospital adverse events and over 3.5 million physician office visits each year are attributable to adverse drug events (5).

The Food and Drug Administration Amendments Act (FDAAA) of 2007 granted FDA authority to require risk evaluation and mitigation strategies (REMS) to ensure that the benefits of a drug outweigh its risks (6). REMS are required risk management plans that use risk minimization strategies beyond the professional product labeling (7). REMS can be required for existing drugs on the market, new drug applications (NDAs), abbreviated NDAs (ANDAs) for generic drugs, and biologics license applications (BLAs) (6). Between enactment of FDAAA and September 2019, 284 REMS programs have been approved by FDA for a wide-range of therapeutic areas affecting the treatment of obesity and diabetes, depression, and pain management (8). Please see Table 1 for definitions of common FDA and REMS terms.

**Table 1:** Common terms and acronyms for US Food and Drug Administration (FDA) and Risk Evaluation and Mitigation Strategies (REMS).

**Terms, acronyms, abbreviations**	**Definition**
**Relevant Legislation**	
Food and Drug Administration Amendments Act (FDAAA)^*^	Law enacted in 2007 reauthorizing and expanding PDUFA, among others, to provide FDA with new authorities to require postmarket studies, safety labeling changes, and REMS
Prescription Drug User Fee Act (PDUFA)^†^	Created by Congress in 1992 to authorize FDA to collect fees from companies producing certain human drugs and biological process to expedite the drug approval process
**Application Types and Submissions**^‡^	
Abbreviated New Drug Application (ANDA)	Vehicle through which drug sponsors formally propose that FDA approve a generic drug product for sale and marketing in the U.S.
Biologics License Application (BLA)	Vehicle through which drug sponsors formally propose that FDA approve a biologic product for sale and marketing in the U.S.
New Drug Application (NDA)	Vehicle through which drug sponsors formally propose that FDA approve a new drug product for sale and marketing in the U.S.
Periodic Safety Update Reports (PSUR)^**^	Documents intended to provide a safety evaluation of the drug product for submission by manufacturers at defined time points during the post marketing phase
**REMS Programs^¶^ and Components^#^**	
REMS	A drug safety program that the FDA can require for certain medications with serious safety concerns to help ensure the benefits of the medication outweigh its risks
Active REMS	Products whose REMS program and requirements are in effect
Released REMS	Products whose REMS program is no longer required by the FDA
Shared System REMS^‖^	REMS programs developed for multiple prescription drug products and implemented jointly by two or more manufacturers
Communication Plan (CP)	Letters, websites, and fact sheets directly to healthcare providers informing of specific safety risks identified in the REMS and steps to take to reduce the risk
Medication Guide (MG)	Handouts for patients distributed with prescription medications that contain FDA-approved information to help inform about how to use a medication and avoid serious adverse events in patient-friendly language
Elements to Assure Safe Use (ETASU)	Required activities such as healthcare provider training, patient counseling and monitoring that support the safe use of the medication

* *FDA. FDAAA Implementation—Highlights One Year After Enactment. Available online at: https://www.fda.gov/regulatory-information/food-and-drug-administration-amendments-act-fdaaa-2007/fdaaa-implementation-highlights-one-year-after-enactment [cited 2020 January 28]*.

** *21 CFR 314.80(c)(2) and 600.80(c)(2)*.

† *FDA. Prescription Drug User Fee Amendments. [cited 2020 January 17]. Available online at: https://www.fda.gov/industry/fda-user-fee-programs/prescription-drug-user-fee-amendments*.

‡ *FDA. Types of Applications. Available online at: https://www.fda.gov/drugs/how-drugs-are-developed-and-approved/types-applications [cited 2020 January 17]*.

¶ *FDA. Approved Risk Evaluation and Mitigation Strategies (REMS). Available online at: https://www.accessdata.fda.gov/scripts/cder/rems/index.cfm [cited 2020 January 17]*.

‖ *FDA. Development of a Shared System REMS: Guidance for Industry. Draft Guidance In: DHHS, editor. (2018)*.

# *FDA. What's in a REMS? Available online at: https://www.fda.gov/drugs/risk-evaluation-and-mitigation-strategies-rems/whats-rems [cited 2020 January 17]*.

Early in the implementation of REMS, the majority of programs included strategies focused on dissemination of risk information. REMS programs may require that drug manufacturers develop materials for patients, such as a Medication Guide, which contain FDA-approved information in patient-friendly language that can help inform patients about how to use a medication and avoid serious adverse events. After guidance issuance in 2012, FDA no longer required every Medication Guide to be part of a REMS, however, they still remain part of the FDA-approved labeling (9). In most cases, FDA includes a Medication Guide as part of a REMS only when the REMS includes other clinical interventions such as patient counseling (10). Other dissemination strategies include targeting healthcare providers; these are known as Communication Plans. REMS may require drug manufacturers to communicate directly to healthcare providers involved in the delivery of health care or medications or develop certain packaging and safe disposal technologies (11, 12). Most of the REMS that included only a Medication Guide or Communication Plan have now been released under the mandate of REMS.

Today, the majority of active REMS programs (84%, 51 out of 61programs) involve complex multi-level interventions (8). In these situations, FDA requires healthcare providers to conduct clinical interventions known as elements to assure safe use (ETASU) that support the safe use of the medication. ETASU may include: training or certification of prescribers, training or certification of dispensers, dispensing/administering the drug in certain settings, requiring evidence or documentation of safe use conditions, monitoring of patients, and/or enrolling patients in a registry (13).

REMS programs, although developed by drug manufactures, are essentially one form of public health intervention programs that need to be implemented within the US healthcare system and adopted by healthcare providers. For example, the Opioid Analgesics REMS program is one strategy among multiple national and state efforts to reduce the risk of abuse, misuse, addiction, overdose, and deaths due to prescription opioid analgesics. It requires that training be made available to all healthcare providers who are involved in the management of patients with pain, including nurses and pharmacists (14). In 2009, the Zyprexa Relprevv REMS was approved to reduce the risk of post-injection delirium sedation syndrome. The REMS was developed to make sure all patients receive special monitoring during the period just following drug administration when post-injection delirium sedation syndrome is most likely to occur, so it can be detected and treated (15).

Drug manufacturers are also required to assess the effectiveness of their REMS program and submit assessment reports to FDA at specified frequency. Manufacturers generally develop a REMS assessment plan prior to approval. The REMS assessment plan is a specific plan for how the drug manufacturer intends to assess the performance of the REMS in meeting its risk mitigation goals and objectives (10). Each assessment plan includes a number of assessment measures to evaluate processes and outcomes. Depending on the complexity of the program, the number of assessment measures may vary. An example of a measure assessing processes may include the number of prescribers, health care settings, and pharmacies that have undergone training in the REMS program. An example of a measure assessing outcomes may include numbers and rates of a specific adverse event of interest such as rates of serious bleeds or severe neutropenia (16). The REMS assessment plan is outlined in the original REMS approval letters for all NDAs and BLAs and is made publicly available through the FDA website, REMS@FDA (also available at DRUGS@FDA).

Assessing the effectiveness of REMS programs is challenging. For example, drug manufacturers and healthcare providers have expressed concerns associated with the challenges of collecting data and lack of standardized format for assessment plans (17). Early following the implementation of REMS, the Office of the Inspector General report raised concerns about the effectiveness of the REMS programs and recommended that FDA should develop and implement a plan to identify, develop, validate, and assess REMS components (2). The report also recommended that FDA should identify and implement reliable methods to assess the effectiveness of REMS.

In response and to modernize post-marketing drug safety, the FDA committed as part of the fifth authorization of the prescription drug user fee program to develop evidence-based methodologies for assessing the effectiveness of REMS (3). The Prescription Drug User Fee Act (PDUFA) gives FDA authority to collect fees from companies that produce drugs when they submit NDA and BLA applications in exchange for ensuring timely review; PDUFA is reauthorized by Congress every 5 years, providing new windows of opportunity for advancing public policy by allowing manufacturers and the FDA to discuss and negotiate commitments to facilitate “timely access to safe, effective, and innovated new medicines for patients.”

In 2019, FDA issued a draft guidance entitled “REMS Assessments: Reporting and Planning” (*henceforth referred to as the Assessment Guidance*) in which it encouraged “applicants and the research community to develop novel methods for assessing REMS” (10). The draft Assessment Guidance outlines five categories for evaluation, including: Outreach and Communications, Implementation and Operations, Knowledge, Safe-Use Behaviors, and Health Outcomes; see Table 2.

**Table 2:** Adaptation of RE-AIM dimensions as applied to REMS assessment measures and Assessment Guidance categories.

**RE-AIM dimension**	General description^*^	Description as applied to REMS assessments^**^	**Assessment guidance category**	**Definitions of assessment guidance category**
Reach	Reach refers to the absolute number, proportion, and representativeness of individuals who are willing to participate in a given initiative, intervention, or program	**Patient (individual level)** • Number of patients treated or enrolled (numerator) • Proportion of eligible patients (“valid denominator” given the drug's indicated use) treated or enrolled • Characteristics of patients treated or enrolled compared with nonparticipants—representativeness	Outreach and Communications	Measures of the extent to which the REMS materials reached the intended stakeholders
Effectiveness	Effectiveness refers to the impact of an intervention on important outcomes, including potential negative effects, quality of life, and economic outcomes	**Patient (individual level)** • Knowledge-Attitudes; Process-Behavior; Health Outcomes and/or Surrogates • Positive and negative (unintended) impacts; observed vs. expected rates of effectiveness • Heterogeneity (variability) of effect across different subpopulations	Safe Use Behaviors and Knowledge Health Outcomes	Measures of the extent to which safe use conditions are being adopted or followed, or of stakeholders' knowledge about the REMS-related risk or knowledge of any safe use conditionsMeasures of the safety-related health outcome of interest or a surrogate of a health outcome
Adoption	Adoption refers to the absolute number, proportion, and representativeness of settings and intervention agents (people who deliver the program) who are willing to initiate the program	**Health Care System (setting level)** • Number of practices, clinics, hospitals or pharmacies certified or enrolled (numerator) • Proportion of eligible practices, clinics, hospitals or pharmacies (“valid denominator” given the drug's indicated use) certified or enrolled • Characteristics of practices, clinics, hospitals or pharmacies certified or enrolled compared with non-adopters—representativeness **Health Care Provider (agent level)** • Number of prescribers and/or pharmacists certified or enrolled (numerator) • Proportion of eligible prescribers and/or pharmacists (“valid denominator” given the drug's indicated use) certified or enrolled • Characteristics of prescribers and/or pharmacists certified or enrolled compared with non-adopters—representativeness	Outreach and Communications	Measures of the extent to which the REMS materials reached the intended stakeholders
Implementation	At the setting level, implementation refers to the intervention agents' fidelity to the various elements of an intervention's protocol, including consistency of delivery as intended and the time and cost of the intervention Implementation elements include: implementation fidelity, adaptation, and cost of intervention At the agent level, implementation refers to the clients' use of the intervention strategies	**Health Care System (setting level)** • Percent of targeted groups who were sent, received REMS information and/or training (by mode and frequency of distribution) • Curriculum consistency—fidelity and adaptation over time (by training modality) • Extent of completed, successful training and/or certification in the program • Incremental costs and resources required (fixed and variable) for REMS participation • Heterogeneity (variability) of implementation across different settings	Implementation and Operations	Measures of the extent to which the intended stakeholders are participating in the program, how effectively the REMS program is being implemented and any unintended consequences such as patient access or burden to the healthcare system
		**Health Care Provider (agent level)** • Educational effectiveness measured by: knowledge-attitudes, behavioral intention for safe use processes and procedures, observed behavior-compliance • Heterogeneity (variability) of implementation across different settings and/or provider characteristics	Safe Use Behaviors and Knowledge	Measures of the extent to which safe use conditions are being adopted or followed, or of stakeholders' knowledge about the REMS-related risk or knowledge of any safe use conditions
Maintenance	At the setting level, maintenance reflects the extent to which the program or processes become institutionalized or sustained as part of routine practice over time	**Health Care System (setting level)** • Cumulative real-world evidence of the integration of REMS processes and procedures into state and institutional policies, treatment guidelines, insurance requirements	Not included	Not applicable
	At the agent or individual level, maintenance reflects the extent to which practices become a stable part of the behavioral repertoire of the individual	**Health Care Provider (agent level) and Patient (individual level)** • Cumulative evidence over time to include: durability of knowledge; compliance with REMS processes and procedures; attrition rate (from the program); heterogeneity (variability) of attrition by subgroups, unintended outcomes, e.g., access or burden issues	Not included	Not applicable

* *Defined in Gaglio et al. (18)*.

** *Informed by the National Cancer Institute (19)*.

Using the RE-AIM (Reach, Effectiveness, Adoption, Implementation, Maintenance) framework may be suitable for evaluating REMS programs. RE-AIM is a framework to enhance the translation of research into practice through the adoption and implementation of evidence-based interventions. The framework was initially used to evaluate prevention and health behavior change programs, and more recently, has been used to help plan programs and improve their chances of working in “real-world” settings. The overall goal of the RE-AIM framework is to encourage program planners, evaluators, researchers, funders, and policymakers to consider essential program elements including external validity. Its five dimensions are designed to enhance the quality, speed, and impact of public health efforts and involve the following: reach of intended target population, effectiveness on important outcomes, adoption by target staff or settings, implementation consistency, and maintenance of intervention effects over time in individuals and settings (20).

RE-AIM addresses all components of REMS programs, including compliance processes, program participation, and overall outcomes, as suggested by the Assessment Guidance (10). Moreover, RE-AIM is an evaluation framework from implementation science that has been widely applied to evaluate health interventions similar to REMS programs (18). For example, RE-AIM has been used by the Centers for Disease Control and Prevention (CDC) for the evaluation of the implementation of the Diabetes Prevention Group (21). Another example includes the application of RE-AM to evaluation of implementing physical activity as a standard of care in healthcare settings (22). Its application has been proposed as an extension to assess the public health impact of policy change (23). The objective of this study was to characterize REMS assessment plans using RE-AIM and to identify areas for advancing methods for evaluating REMS programs.

## Methods

**Figure 1:**
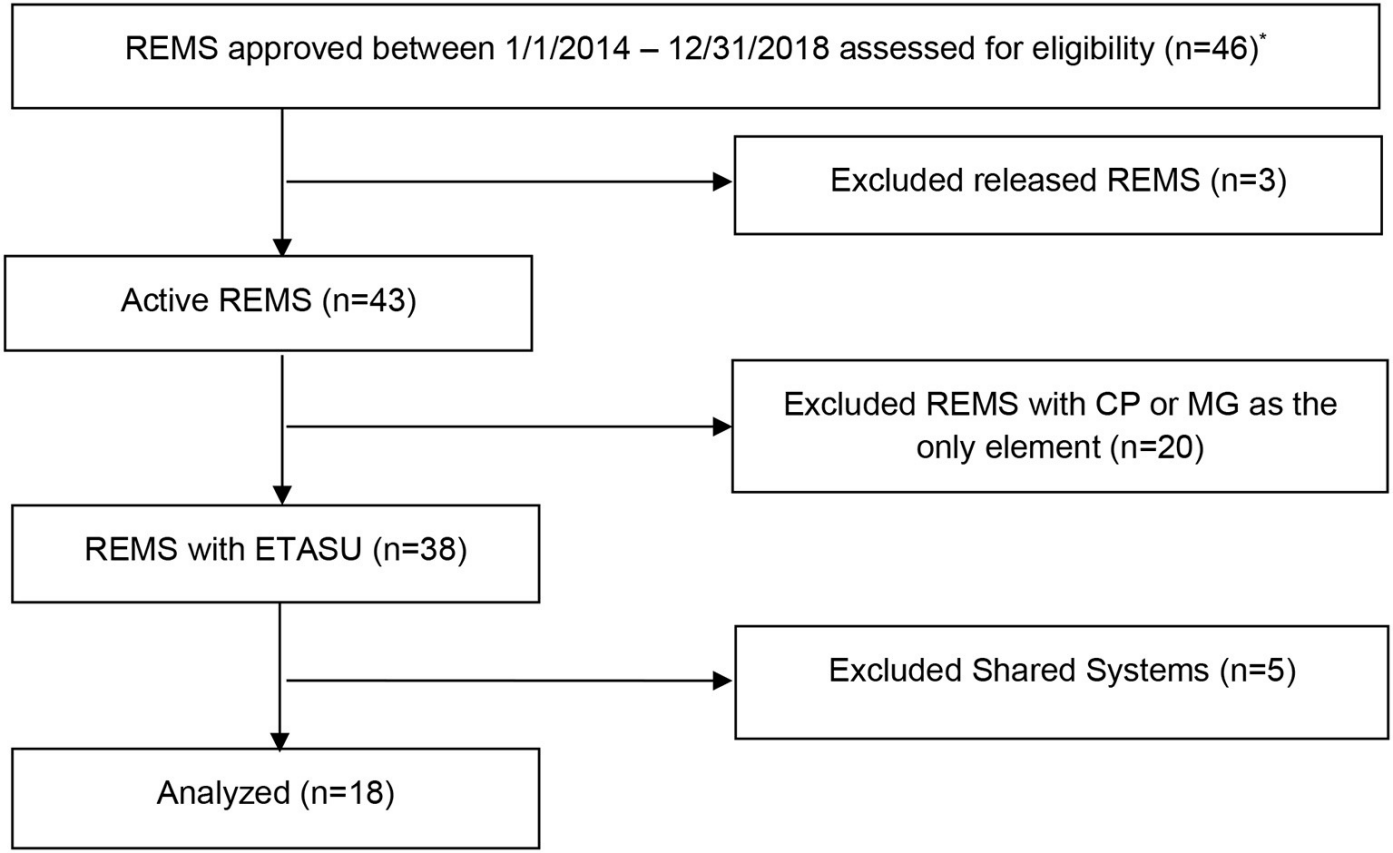
Flow diagram of 2014–2018 active, single product REMS with ETASU program selection for content analysis of assessment plans using the RE-AIM framework. ANDA, Abbreviated New Drug Application; CP, Communication Plan; MG, Medication Guide; *REMS programs were accessed for eligibility January 2019.

A content analysis of REMS assessment plans (*N* = 18) and measures(*n* = 540) was conducted for REMS programs approved by the FDA between January 1, 2014 and December 31, 2018. Given that the first REMS was approved in 2008, we limited our study sample to REMS programs approved in the past 5 years as these would be more aligned with current policy. Programs were excluded if they had been released during this timeframe and were no longer required by the FDA to be implemented. We also excluded REMS containing Medication Guides and Communication Plans as the sole elements because we wanted to study complex multi-level, multi-system interventions, leaving active REMS with ETASU for analysis. Finally, shared system REMS were excluded because we wanted to focus on new programs, and shared system REMS programs reflect sustaining programs that have been adapted for generic products. Figure 1 shows the selection process.

### Source Data

The assessment plan for each REMS program was obtained from the publicly-available regulatory approval letters downloaded from the FDA's website REMS@FDA on January 15, 2019. Assessment plans include a listing of measures that drug manufacturers need to address in their scheduled assessment reports, often at 6-, 12-month, and annually. Each assessment plan is tailored to each REMS program which results in variability in number and type of measures assessed per program. The original approval letters represent measures pre-specified at the time of approval.

### Adaptation of the RE-AIM Dimensions

Using the established RE-AIM framework (20, 21), the authors Toyserkani (GT), Huynh (LH), and Morrato (EM) created construct definitions applicable to REMS assessments by adapting from those defined by the framework as shown in Table 2.

The adaptation for applying RE-AIM to assessment of REMS was informed by the Scoring Instrument developed for assessing NCI Research-Tested Intervention (RTIPs) programs (19). RTIPs is a searchable database of evidence-based cancer control interventions and program materials and is designed to provide program planners and public health practitioners easy and immediate access to research-tested materials. The adaptation was also informed by the RE-AIM checklist for “RE-AIM Dimensions When Evaluating Health Promotion Programs and Policies” found at RE-AIM.org (24).

The goal was to be as consistent with the constitutive definitions of the RE-AIM dimensions as possible. For example, Adoption was defined as the number and proportion of healthcare settings and providers that agree to initiate program or policy change and how representative they are of the intended audience in terms of the setting and the staff. As REMS programs are multi-level interventions, dimensions were further delineated based on the healthcare setting (system level), healthcare provider (agent level), and patient (individual level).

The dimensions were then mapped to categories outlined in the Assessment Guidance to discern the ease of mapping RE-AIM to the Guidance and determine where opportunities in the assessment process may exist.

### Coding

Three blinded reviewers (GT, LH, EM) adjudicated the application of RE-AIM dimensions by coding each REMS assessment measure for three randomly selected REMS assessment plans (average IRR = 75%). They discussed coding discrepancies and refined the dimensions' definitions accordingly. Two blinded reviewers (GT, LH) then categorized each assessment measure(*n* = 540) for the remaining 15 assessment plans with a third reviewer (EM) serving as an adjudicator.

### Analysis

Sensitivity analyses were performed to examine qualitative differences over time, by type of application (NDA vs. BLA), and by type of ETASU required. Descriptive statistics were calculated to determine the proportions of RE-AIM dimensions per REMS assessment program. The median number of assessment measures were then independently analyzed by the variables: year approved, application type, and ETASU to identify any correlations.

Using the alignment between RE-AIM dimensions and Assessment Guidance categories, each assessment measure was then assessed for its inclusion of the categories. This was done by noting how many assessment measures were reflective of each category and then measuring these individual values against the total number of assessment measures for each program. Aggregate summary statistics were reported for the number of measures per category and frequency distribution across all programs.

## Results

A total of 18 REMS programs involving nine NDAs and nine BLAs met evaluation eligibility criteria. Table 3 shows the characteristics of the REMS programs meeting criteria at the time of their original REMS approval. Programs by year approved ranged from two in 2016 to five in 2018. The drug products carried a variety of risks intended to be mitigated by the REMS, ranging from cancers such as lymphoma and osteosarcoma, immune system disorders such as autoimmune conditions and cytokine release syndrome, and psychiatric disorders such as suicidal ideation and behavior. The number of assessment measures per program ranged from 10 to 57.

**Table 3:** Characteristics of selected active, single product REMS with ETASU programs at time of original approval (2014–2018) included for content analysis of assessment plans using the RE-AIM framework.

**Year**	Drug^**^ (active ingredient)	**Type**	ETASU^***^	**Indication (benefit)**	**Risk(s) requiring risk mitigation**	**Number of assessment measures**
2014	Myalept (*metreleptin*)	BLA	A, B, D	Treat the complications of leptin deficiency in patients with congenital or acquired generalized lipodystrophy	Lymphoma and anti-metreleptin antibodies that neutralize endogenous leptin and/or Myalept	26
	Aveed (*testosterone undecanoate*)	NDA	A, B, C	Testosterone replacement therapy in adult males for conditions associated with a deficiency or absence of endogenous testosterone	Anaphylaxis and pulmonary oil microembolism	22
	Lemtrada (*alemtuzumab*)	BLA	A, B, C, D (CP)	Treatment of patients with relapsing forms of multiple sclerosis	Autoimmune conditions, infusion reactions, and malignancies	36
2015	Natpara (*parathyroid hormone*)	BLA	A, B, D	An adjunct to calcium and vitamin D to control hypocalcemia in patients with hypoparathyroidism	Osteosarcoma	21
	Xyrem (*Sodium oxybate*)	NDA	A, B, D (MG)	Treatment of cataplexy or excessive daytime sleepiness in patients 7 years of age and older with narcolepsy	Serious adverse outcomes resulting from inappropriate prescribing, misuse, abuse, and diversion	57
	Ionsys (*fentanyl iontophoretic) transdermal system*)	NDA	B, C	Short-term management of acute postoperative pain severe enough to require an opioid analgesic in the hospital and for which alternative treatments are inadequate	Respiratory depression resulting from accidental exposure	29
	Addyi (*flibanserin*)	NDA	A, B	Treatment of premenopausal women with acquired, generalized hypoactive sexual desire disorder, as characterized by low sexual desire that causes marked distress or interpersonal difficulty	Hypotension and syncope due to interaction with alcohol	32
2016	Probuphine (*buprenorphine hydrochloride*)	NDA	A, B, C, E (MG)	Maintenance treatment of opioid dependence in patients who have achieved and sustained prolonged clinical stability on low-to-moderate doses of a transmucosal buprenorphine-containing product	Migration, protrusion, expulsion and nerve damage associated with insertion and removal and accidental overdose, misuse and abuse	22
	Zinbryta (*daclizumab*)	BLA	A, B, D, E, F (CP)	Treatment of adult patients with relapsing forms of multiple sclerosis	Hepatic injury and immune mediated disorders	27
2017	Siliq (*brodalumab*)	BLA	A, B, D	Treatment of moderate to severe plaque psoriasis in adult patients who are candidates for systemic therapy or phototherapy and have failed to respond or have lost response to other systemic therapies	Suicidal ideation and behavior, including completed suicides	31
	Kymriah (*tisagenlecleucel*)	BLA	B, C	Treatment of: Pediatric and Young Adult Relapsed or Refractory (r/r) B-cell Acute Lymphoblastic Leukemia and Adult Relapsed or Refractory (r/r) Diffuse Large B-Cell Lymphoma	Cytokine release syndrome and neurological toxicities	21
	Yescarta (*axicabtagene ciloleucel*)	BLA	B, C	Treatment of adult patients with relapsed or refractory large B-cell lymphoma after two or more lines of systemic therapy	Cytokine release syndrome and neurological toxicities	21
	Sublocade (*buprenorphine extended-release*)	NDA	B	Treatment of moderate to severe opioid use disorder in patients who have initiated treatment with a transmucosal buprenorphine-containing product, followed by dose adjustment for a minimum of 7 days	Intravenous self-administration	20
2018	Jynarque (*tolvaptan*)	NDA	A, B, D, E, F (CP)	Slow kidney function decline in adults at risk of rapidly progressing autosomal dominant polycystic kidney disease	Liver injury	42
	Palynziq (*pegvaliase-pqpz*)	BLA	A, B, D	Reduce blood phenylalanine concentrations in adult patients with phenylketonuria who have uncontrolled blood phenylalanine concentrations greater than 600 micromol/L on existing management	Anaphylaxis	31
	Tegsedi (*Inotersen*)	NDA	A, B, D, E, F	Treatment of polyneuropathy of hereditary transthyretin-mediated amyloidosis in adults	Bleeding with thrombocytopenia and glomerulonephritis	57
	Dsuvia (*sufentanil*)	NDA	B, C	Use in adults in certified medically supervised healthcare settings for the management of acute pain severe enough to require an opioid analgesic and for which alternative treatments are inadequate	Respiratory depression from accidental exposure	35
	Ultomiris (*ravulizumab-cwvz*)	BLA	A	Treatment of adult patients with paroxysmal nocturnal hemoglobinuria	Meningococcal infections	10

*BLA, Biologic License Application; NDA, New Drug Application; ETASU, Elements to Assure Safe Use; CP, Communication Plan; MG, Medication Guide*.

** *REMS programs were selected January 2019*.

*** *ETASU A, training or certification of prescribers; ETASU B, training or certification of dispensers; ETASU C,dispensing/administering the drug in certain settings; ETASU D, requiring evidence or documentation of safe use conditions; ETASU E, monitoring of patients; ETASU F, enrolling patients in a registry*.

### Frequency Distribution Analysis of RE-AIM Dimensions

Table 4 shows the distribution of REMS programs (*N* = 18) and assessments measures (*n* = 540) across the RE-AIM dimensions. The 18 programs yielded a total of 540 assessment measures; of these, only three measures (0.6%) could not be mapped to a single RE-AIM dimension. These included measures where the intent was unclear or there were multiple intents of the assessment measure that it could have been categorized into more than one dimension.

**Table 4:** Distribution of REMS programs and assessment measures across RE-AIM dimensions.

	**Programs Addressing the RE-AIM Dimension (*N*, percentage of total)**	**Assessment measures per REMS program (median, range)**	**Assessment Measures (*n*, percentage of total)**
Total Sample	18 programs	31 (range 10–57)	537 assessment measures^*^
**RE-AIM Dimension**			
Reach	15 (83.3%)	2 (range 0–7)	48 (8.9%)
Effectiveness	16 (88.9%)	2.5 (range 0–8)	49 (9.1%)
Adoption	18 (100%)	3.5 (range 0–7)	61 (11.4%)
Implementation	18 (100%)	18 (range 4–41)	371 (69.6%)
Maintenance	8 (44.4%)	0 (range 0–1)	(1.5%)

* *3 measures (0.6%) could not be mapped to a single RE-AIM dimension*.

Of 18 REMS programs, the median number of assessment measures assessing Reach per assessment plan was 2 (IQR: 2–4). Prototypical examples of REMS assessment measures categorized as assessing reach included “age and gender of enrolled patients” and “total number of orders shipped to pharmacies.”

Similarly, the median number of assessment measures assessing Effectiveness per REMS assessment plan was 2.5 (IQR: 1–4). Prototypical examples of REMS assessment measures categorized as assessing effectiveness included “adverse event assessments” and “an evaluation of knowledge of patients of the increased risks.”

Regarding Adoption, the median number of assessment measures assessing this dimension per REMS assessment plan was 3.5 (IQR: 2–5). Prototypical examples of REMS assessment measures categorized as assessing adoption included the “number of newly enrolled and active pharmacies stratified by type of pharmacy and geographic location” and “number and location of REMS training programs.”

The median number of assessment measures assessing Implementation per REMS assessment plan was 18 (IQR: 15–24). Prototypical examples of REMS assessment measures categorized as assessing implementation included: “date when the REMS website went live,” “summary report of program problems reported and corrective actions resulting from issues identified,” and “number of prescriptions written by non-certified prescribers and detailed root-cause analysis.”

Finally, the median number of assessment measures assessing Maintenance per REMS assessment plan was 0 (IQR: 0–1). Prototypical examples of REMS assessment measures categorized as assessing maintenance included: “number of discontinued patients” and “number of healthcare settings re-enrollments and the expected number of re-enrollments.”

**Figure 2:**
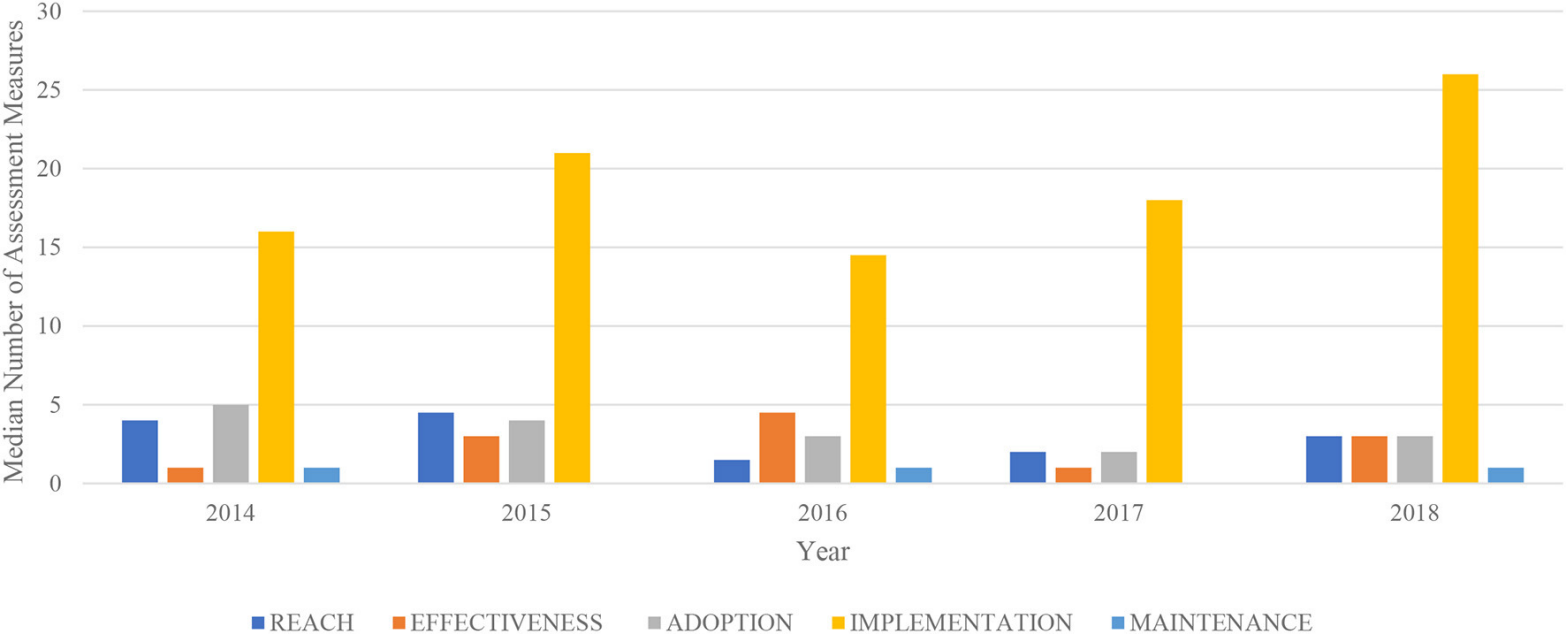
Median number of REMS assessment measures per RE-AIM dimension by year (2014–2018).

Figure 2 shows a lack of time trends in RE-AIM dimensions by year of REMS approval. No trends in the number or distribution of RE-AIM dimensions were observed by drug application type or specific ETASU element required.

### Alignment With FDA Assessment Guidance

Consistent with the adaptation of RE-AIM to REMS, the application of RE-AIM dimensions to the Assessment Guidance demonstrated heavy focus on Implementation and Operations. Because assessment measures categorized into the Implementation dimension could be measuring either the healthcare provider's knowledge or processes, these were categorized as either Implementation and Operations (*n* = 315, 58%) or Safe Use Behaviors and Knowledge (*n* = 52, 10%) according to the Assessment Guidance. Likewise, measures categorized into the Effectiveness dimension were categorized as Safe Use Behaviors and Knowledge (*n* = 20, 4%) if they assessed knowledge, and Health Outcomes (*n* = 30, 5%) if they measured patient understanding. Outreach and Communications (*n* = 112, 22%) were akin to Reach or Adoption depending on the target audience. Measures of maintenance (*n* = 8, 1%) were lacking in REMS assessments.

## Discussion

To our knowledge, this is the first systematic content analysis examining the feasibility and utility of applying an implementation science framework across a range of REMS programs. The application of social science theories and frameworks to pharmaceutical risk minimization design, implementation and evaluation has been discussed by Smith and Morrato (25). Others have proposed a variety of implementation measures for health services research and pharmaceutical risk minimization evaluation (26–28). Theories and frameworks can provide a social science mechanism of action to understand the relationship between measures and the causal pathway affecting the success of REMS programs in much the same way that a biological mechanism of action guides the clinical development of new medicines. Even risk minimization programs that address only a subset of constructs with a theoretical model can be framed conceptually, so that regulators and the public perceive the larger context and body of literature guiding these programs (29). Ultimately, the use of theories and frameworks helps enable cross-program comparisons and foster generalizable knowledge to advance the science of risk mitigation dissemination and implementation.

Our pragmatic application of RE-AIM to REMS assessment plans were feasible and relatively intuitive to perform. The primary challenge was defining who is the program recipient and who is the program agent, given the complex and multi-faceted nature of REMS programs and their systems-, provider- and patient-level involvement. As Glasgow et al. have defined the product of Reach and Effectiveness to be the individual level, we interpreted this to refer to patients—the recipient of the program or “groups receiving” the intervention (21). Adoption and Implementation then corresponded to the agents of the program, or “staff members” and “program-level” participants who facilitate the delivery of the program to the patients. Glasgow identified the product of these two dimensions to be at the organization level, which we construed to apply to the healthcare providers and healthcare settings. Once we established this distinction of program recipient vs. agent, determining the RE-AIM dimension of each REMS assessment measure was intuitive.

Our findings demonstrate strong congruence between the RE-AIM framework and REMS assessment measures. Consistent with the Assessment Guidance statement that “REMS can be assessed using both process indicators and the intended outcomes,” application of RE-AIM to REMS assessment plans found heavy emphasis on Implementation and Operations measures (10). However, this research also detected lighter emphasis on Health Outcomes measures in REMS assessment plans. Health outcomes in general are difficult to assess especially given rare adverse drug events. For the majority of drugs, FDA relies on routine pharmacovigilance and spontaneous adverse event reporting(passive surveillance) received through post-market periodic safety update reports (PSURs) (30). However, under-reporting is a major drawback and may underestimate the number of adverse drug events (30, 31). In certain REMS programs, FDA does require patient registries, such as pregnancy registry for drugs with risk of birth defects, that collect case data on safety events (active surveillance) (32). Given that health outcomes are generally challenging to assess, assessment of REMS program effectiveness typically relies on process measures such as knowledge attainment and safe use behaviors.

This research also observed very low inclusion of Maintenance measures in REMS assessment plans. Adding measures of Maintenance to REMS assessment plans can strengthen the quality of REMS programs. Maintenance measures represent an area for real-world evidence of REMS integration into the healthcare system and its sustainability. For example, these metrics would measure the durability of knowledge of healthcare providers or cumulative enrollment of healthcare providers in a program over time or evaluate for the evidence of the integration of REMS processes and procedures into state and institutional policies, treatment guidelines, insurance requirements. Our findings are very similar to conclusions reached by previous studies citing that maintenance and representativeness were reported much less often in other health intervention evaluations (33).

The RE-AIM framework offers a number of strengths, including the fact that it considers representativeness and characteristics of the participants to assess heterogeneity of impact. By assessing heterogeneity of impact, RE-AIM permits evaluations of patient access, healthcare system, and patient burden, a potential unintended consequence of risk mitigation requirements that is of public stakeholder interest. RE-AIM also addresses the often-neglected goal of long-term maintenance at both the individual and organization levels. Finally, RE-AIM considers both process and outcome measures, covering the scope of many domains of interest for REMS.

Another important strength is using frameworks like RE-AIM can help REMS assessments be more transparent and better understood across all stakeholders, as it was originally intended. Having a framework for evaluating REMS can facilitate standardization, consistency, and completeness in assessing REMS to enable comparisons across programs (33). By using the commonly recognized constructs and terminology of RE-AIM, data collected by the REMS program can be more meaningful.

Our application of RE-AIM demonstrated some challenges of the framework. The first challenge is one of definition. It has been acknowledged that RE-AIM application has “frequent issues with confusing different dimensions” (33). For example, as aforementioned, defining the agent and recipient of the program is open to interpretation. Others suggest defining Reach at the healthcare provider-level, not the patient-level as we did (34). Effectiveness has been applied to non-patient stakeholders, such as the healthcare provider and pharmacists, at times “requir[ing] multiple creative and innovative combinations of metrics” (34, 35). This contrasts our interpretation of Effectiveness to apply only at the patient-level, which, as aforementioned, was to be as consistent with the constitutive definition of RE-AIM as possible (24).

The second challenge is one of longitudinal assessment. It is not readily apparent how best to apply the framework in a longitudinal and time-dependent manner, although RE-AIM has been proposed for evaluating adaptation over time (33). Pharmaceutical risk management consists of the iterative process of assessing a product's risk-benefit balance, developing and implementing tools, evaluating the tools, and making adjustments to maintain or improve the benefit-risk balance (36). A single REMS program may be implemented for decades as long as the drug product remains on the market. Moreover, the FDAAA requires that assessments be conducted at 18 months, 3 years, and 7 years post-market, at a minimum (13). FDA has required more frequent assessments for REMS with ETASU. Further research is needed to elucidate the pragmatic use of the framework during the REMS life cycle by aligning and differentiating specific RE-AIM measures at different time points of adoption. For example, what are early markers of Effectiveness vs. later markers? What are early markers of Maintenance vs. later markers?

The third challenge is one of utility for decision making. Regulators need to use assessment data to determine whether to sustain, modify or eliminate a REMS program. Should a REMS regulatory determination require a collective gestalt of all RE-AIM dimensions or rely on a single dimension, and if so, how might that best be accomplished in a standardized manner? Our research, similar to previous applications of RE-AIM, found that not all dimensions were assessed equally (18). This observation raises the question of whether all five dimensions are of equal importance, or are there dimensions that are more important, when determining whether a REMS program is meeting its public health drug safety goals.

The limitations of our study include examining only the RE-AIM framework to characterize REMS assessment plans. Future work should evaluate the application of other established frameworks such as PRECEDE-PROCEED, Consolidated Framework for Implementation Research (CFIR), and Practical, Robust Implementation Sustainability Model (PRISM) (33, 37–39). Secondly, this study looked at REMS assessment plans from 2014 to 2018 and does not consider the potential impact or evolution of REMS assessment plans since the publication of the Assessment Guidance issued in 2019. Furthermore, assessment measures from Shared Systems REMS (multiple products of the same class or molecular moiety under two or more sponsors) can offer additional insights into the strengths and opportunities for REMS assessments.

Of note, our study examined the type and quantity of REMS assessment measures from the original approval; however, it did not assess the rigor of proposed study designs nor the quality of their reports. Reporting standards for risk minimization communication and program evaluation have been described by members of the International Society for Pharmacoepidemiology (40). A systematic review of the published literature on pharmaceutical risk minimization evaluation found limited use of conceptual frameworks guiding process and outcome measurement selection and program design and implementation (41).

FDA considers public comments and stakeholder feedback as it finalizes guidance to industry. Therefore, learning from the current analysis is one source of input that FDA is considering as it works on the final version of the REMS Assessment Guidance affecting all future REMS programs. The guidance aims to ultimately improve how REMS assessment plans are developed, specifically how the REMS program goals, objectives and REMS design may impact the selection of metrics and data sources, which will be used to assess whether the program is meeting its risk mitigation goals (16).

In addition, findings are also relevant to FDA's efforts on structured benefit-risk assessment process and commitments established in the sixth authorization of the PDUFA VI in 2017. FDA has made several commitments in PDUFA VI for continued implementation of structured benefit-risk assessment, including publishing a draft guidance on benefit-risk assessment. “Risk and Risk Management” is one explicit dimension in the benefit-risk assessment framework (42). How information, including evidence and uncertainties, can be effectively communicated to the public is one area of interest. To meet requirements established in the 21st Century Cures Act, the benefit-risk assessment guidance will also discuss how relevant patient experience data may be used to inform benefit-risk assessments (43). RE-AIM provides a natural structured analytic approach for synthesizing risk management effectiveness evidence and integrating patient data into the assessment.

## Conclusion

Dissemination and implementation science frameworks can provide a systematic approach for REMS program assessments. They provide a structured and evidence-based approach to guide what should be evaluated and to what extent, and to identify which aspects of the programs will be considered when judging REMS program performance, including *a priori* expectations for program success. Frameworks like RE-AIM, can be readily applied to REMS assessments to strengthen their evaluation and have the potential to advance science, quality of practice, and population health through all participants affected by REMS.

## Data Availability Statement

The datasets generated for this study are available on request to the corresponding author.

## Author Contributions

All authors listed have made a substantial, direct and intellectual contribution to the work, and approved it for publication.

### Conflict of Interest

The authors declare that the research was conducted in the absence of any commercial or financial relationships that could be construed as a potential conflict of interest.
